# HIV treatment and care among Italian inmates: a one-month point survey

**DOI:** 10.1186/s12879-015-1301-5

**Published:** 2015-12-10

**Authors:** R. Monarca, G. Madeddu, R. Ranieri, S. Carbonara, G. Leo, M. Sardo, F. Choroma, S. Casari, D. Marri, A. A. Muredda, F. A. Nava, S. Babudieri, SIMSPe–SIMIT Group

**Affiliations:** Infectious Diseases Unit, Belcolle Hospital, Viterbo, Italy; Infectious Diseases Unit, Department of Clinical and Experimental Medicine, University of Sassari, Viale San Pietro 35b, 07100 Sassari, Italy; Infectious Diseases Unit, A.O. San Paolo, Milan, Italy; Institute of Infectious Diseases, University of Bari, Bari, Italy; Infectious Diseases Unit, Amedeo di Savoia Hospital, Torino, Italy; Cotugno Hospital, Infectious Diseases Unit, Naples, Italy; Infectious Diseases Unit, AUSL, Parma, Italy; Institute of Infectious Diseases, University of Brescia, Brescia, Italy; Infectious Diseases Unit, A.O. Santa Maria alle Scotte, Siena, Italy; Penitentiary Medicine, Padova, Italy

**Keywords:** HIV, Patient care, Screening, Antiretroviral treatment, Medication adherence

## Abstract

**Background:**

HIV infection, with an estimated prevalence be between 2 and 50 times those of the general adult population is a major health challenge for prison authorities worldwide. Since no nationwide surveillance system is present in Italy, data on HIV prevalence and treatment in prisons are limited to only a few and small observational studies. We aimed to estimate HIV prevalence and obtain an overview on diagnostic and therapeutic activities concerning HIV infection in the Italian penitentiary system.

**Methods:**

We piloted a multi-centre cross-sectional study investigating the prevalence of HIV infection and assessing HIV-related medical activities in Italian correctional institutions.

**Results:**

A total of 15,675 prisoners from 25 institutions, accounting for approximately one-fourth of the prison inmates in Italy, were included in the study, of whom, 97.7 % were males, 37.1 % foreigners and 27 % had a history of intravenous drug addiction. HIV-tests were available in 42.3 % of the total population, with a known HIV Infection proportion of 5.1 %. In the month prior to the study, 604 of the 1,764 subjects who entered prison were tested for HIV, with a HIV-positive prevalence of 3.3 %. Among the 338 HIV-positive prisoners, 81.4 % were under antiretroviral treatment and 73.5 % showed undetectable HIV-RNA. In 23/338 (6.8 %) a coinfection with HBV and in 189/338 (55.9 %) with HCV was also present. Among the 67 (19.8 %) inmates with HIV who did not receive HIV treatment, 13 (19.5 %) had T-CD4+ count <350 cells/mm^3^ and 9 (69.2 %) of these had refused the treatment. The majority of the inmates with HIV-infection were on a PI-based (62.5 %) or on NNRTIs-based (24.4 %) regimen. Only a minority of patients received once daily regimens (17.2 %).

**Conclusions:**

Although clinical and therapeutic management of HIV infection remains difficult in Italian prisons, diagnostics, treatment and care were offered to the majority of HIV-infected inmates. Specific programs should be directed towards the prison population and strict cooperation between prison and health institutions is needed to increase HIV treatment.

## Background

HIV is a major health challenge for prison authorities worldwide. HIV prevalence within prisons is estimated to be between 2 and 50 times those of general adult populations. Available studies show an HIV prevalence ranging from 0.6 % in the UK to 6 % in Spain and 7.2 % in Italy [1–7].

In Italy measures promoting HIV prevention and control are listed among the health objectives that must be guaranteed to the prison population. HIV tests cannot take place without the consent of the person concerned and may be recommended but not imposed on inmates who display high risk behaviors. Detainees should be informed with all relevant information concerning HIV prevention. Counselling, treatment, care and support services should be part of a comprehensive HIV program aimed at improving health care in prison and at making it equivalent to that available in the community [8–10]. Nowadays, however, these objectives are largely unmet due to the limitations of the Italian penitentiary system, such as severe budget constraints leading to under-resourced prison settings, prison overcrowding, poor condition of the existing health facilities, undertrained and underpaid prison staffing, frequent inmate transfer between prisons or prison wings, court attendance and hospital visits, lack of a national correctional healthcare database and especially the low offering and execution of HIV screening [9–11].

Moreover, the data on treatment of HIV infection in prisons are limited to only a few observational studies, thus undermining current policy for improving prison health care system [10–14].

The aims of this study were firstly, to evaluate the prevalence of HIV infection among new entry inmates, secondly, to evaluate the number of HIV infected inmates who were actually treated and thirdly to describe the antiretroviral treatments offered and report the reasons for lack of treatment.

## Methods

A multi-centre cross-sectional survey was carried out from 1 to 30 July 2013 to investigate the prevalence of HIV infection and to assess HIV-related diagnostic and therapeutic activities in Italian correctional institutions. The prevalence of HIV infection was estimated in the imprisoned population on July 31, 2013. Data on HIV testing was evaluated both in the entire prison population and in the prison entrants during the study period. A questionnaire was emailed to the 206 Italian prisons and was voluntarily completed by health professionals based in the prison establishments using information in medical records. The following data was recorded only for inmates who were present during the study period: general data (sex, ethnicity and drug use), HIV data (HIV testing, viro-immunological profile, visit schedule), HIV treatment data (setting, method, frequency of drug dispensation, type of antiretrovirals used, medication refusal). Returned surveys were collected and consolidated using build-in functions of Microsoft Excel. Where gaps in the questionnaire data remained, prison administrative bodies and experts were contacted via e-mail or telephone and requests for specific information were made.

All studies regarding the Italian Penitentiary System have been nationally approved by the Ethics Committee of the University of Rome Tor Vergata (Registro Sperimentazioni 73/05). No specific consent was required since data were collected in anonymous and aggregate form.

### Statistical analysis

Data was analyzed using the SPSS statistical software, version 6.0. Results are expressed as proportions. When applicable, a two-tailed hypothesis testing for difference in proportions was used (Proportion Test); a *p* value of <0.05 was considered significant.

## Results

### Prison population in Italy

On 31 July 2013 Italian prisons counted 64,873 detainees, almost one per 1,000 inhabitants. Foreign detainees were 22,744. It is worth noting that foreign prisoners had more than doubled between 1990 (15 %) and 2013 (35 %). At the time of the study, Italy’s prisons were the most crowded in the European Union with occupancy at more than 142 % of capacity.

### Survey population

On July 31, a total of 15,675 prisoners (15,318 males and 357 females) were included in the survey, accounting for 24.2 % of the total Italian inmates. Thirty-five of the 206 correctional institutions in Italy from 16 of the 20 Italian regions voluntarily answered the questionnaire: 12 in Northern Italy (*n* = 6,527 inmates), 11 in Central Italy (*n* = 3,846 inmates) and 12 in Southern Italy (*n* = 5,302 inmates). The number of prisoners included in the survey ranged from 7.8 % to 69.1 % according to geographical area. Overall, the institutes in Northern Italy accounted for nearly one-third (32.7 %) of the prisoners included in the survey, compared with 21.5 % in Central Italy and 22.7 % in Southern Italy. During the month prior to the survey 1,764 adults were imprisoned. Table 1 shows the characteristics of inmates entered into the study, according to Italian region. The majority of prisoners were men (97.7 %) with native born Italians accounting for 62.9 % of inmates and foreigners 37.1 % (range 2.0 % - 88.0 %). The vast majority of foreign prisoners included individuals from Morocco (19.0 %), Romania (15.8 %), Albania (12.5 %), Tunisia (12.1 %), Nigeria (4.2 %), Algeria (2.6 %), Egypt (2.0 %) and Senegal (1.8 %). Just under one-third of prison inmates (27.0 %) had a history of heroin or cocaine misuse.

**Table 1:** Total population, number, gender, drug history and foreigners of inmates participating in the survey according to correctional facility region

Region	Number of institutes	Institutes participating in the survey	Total prisoners on July 31	Prisoners participating in the survey	Male prisoners	History of drug misuse	Foreigners participating in the survey
Abruzzo	7	0	2047	0	0	0	0
Basilicata	3	0	483	0	0	0	0
Calabria	12	2	2651	744 (36.3 %)	696 (93.5 %)	105 (14.1 %)	161 (21.6 %)
Campania	17	4	7999	3230 (40.4 %)	3230 (100 %)	867 (26.8 %)	422 (13.1 %)
Emilia Romagna	13	5	3759	1570 (41.8 %)	1507 (100 %)	545 (36.2 %)	861 (54.8 %)
Friuli Venezia Giulia	5	1	845	275 (32.5 %)	275 (100 %)	59 (21.4 %)	172 (62.5 %)
Lazio	14	1	7175	739 (10.3 %)	739 (100 %)	197 (26.6 %)	259 (35.0 %)
Liguria	7	1	1764	240 (13.6 %)	240 (100 %)	100 (41.7 %)	105 (43.8 %)
Lombardia	19	3	8961	1869 (20.8 %)	1868 (99.9 %)	633 (33.9 %)	644 (34.5 %)
Marche	7	1	1108	300 (27.1 %)	279 (93.0 %)	63 (21.0 %)	138 (46.0 %)
Molise	3	0	505	0	0	0	0
Piemonte	13	2	4870	1760 (36.1 %)	1,660 (94.3 %)	346 (19.6)	694 (39.4 %)
Puglia	12	1	4039	428 (10.6 %)	408 (95.3 %)	94 (21.9 %)	120 (28.0 %)
Sardegna	12	1	2095	165 (7.8 %)	148 (89.6 %)	78 (47.2 %)	44 (26.6 %)
Sicilia	26	3	6976	735 (10.5 %)	735 (100 %)	161 (21.9 %)	130 (17.7 %)
Toscana	18	3	4135	733 (17.7 %)	733 (100 %)	232 (31.6 %)	271 (37.0 %)
Trentino Alto Adige	3	1	405	280 (69.1 %)	260 (92.8 %)	50 (17.8 %)	224 (80.0 %)
Umbria	4	2	1672	504 (30.1 %)	504 (100 %)	120 (23.8 %)	201 (39.9 %)
Valle d’Aosta	1	0	278	0	0	0	0
Veneto	10	4	3106	2103 (67.7 %)	2036 (96.8 %)	582 (27.7 %)	1377 (65.5 %)
National Total	206	35	64,873	15,675 (24.2 %)	15,318 (97.7 %)	4232 (27.0 %)	5761 (36.7 %)

**Table 2:** Antiretroviral regimens received by the 275 HIV-infected inmates on ART

ANTIRETROVIRAL DRUGS	n (%)
Protease inhibitors (PI)	
Atazanavir	88 (32.0 %)
Lopinavir	51 (18.5 %)
Darunavir	23 (8.4 %)
Fosamprenavir	9 (3.3 %)
Saquinavir	1 (0.4)
Total	172 (62.6 %)
Non nucleoside reverse trascriptase inhibitors (NNRTI)	
Efavirenz	50 (18.2 %)
Nevirapine	9 (3.3 %)
Rilpivirine	8 (2.9 %)
Etravirine	5 (1.8 %)
Total	72 (26.2 %)
3 Nucleoside reverse transriptase inhibitors (NRTI)	
Abacavir-Lamivudine-Zidovudine	6 (2.2 %)
Integrase inhibitors (INI)	
Raltegravir	24 (8.7 %)
CCR5 inhibitors	
Maraviroc	1 (0.4 %)
NRTI backbone	
Tenofovir disoproxilfumarate-emtricitabine	194 (70.5 %)
Abacavir-lamivudine	41 (14.9 %)
Zidovudine-lamivudine	12 (4.4 %)
Other	28 (10.2 %)

### HIV and other infectious diseases testing

Among the participating penitentiary institutions, 35/35 (100 %) performed screening tests for HIV, HBV and HCV, 27/35 (77.1 %) for tuberculosis and 31/35 (88.6 %) for syphilis.

A voluntary HIV screening, as part of routine clinical evaluations, was available in 6,630 (42.3 %) of the total population at the time of the study and 338 (5.1 %), 330 (97.6 %) males and 8 (2.4 %) females, of these inmates were HIV-infected. Among the 338 HIV-infected inmates 266/338 (78.7 %) were Italian, 72/338 (21.3 %) were foreigners, 23/338 (6.8 %) were also HBsAg-positive and 189/388 (55.9 %) had anti-HCV antibodies.

Regarding risk factors for HIV infection, 175/338 (51.7 %) reported being intravenous drug users (IVDU), 16/338 (4.7 %) were men having sex with men (MSM) and 147/338 (43.5 %) declared sexual contact risk. Furthermore, 85/163 (52.1 %) of non IVDU reported tattooing or body piercing with unsterile equipment. When considering only the 72 HIV-infected foreigners, 49/72 (68.0 %) reported being IVDU, 20/72 (27.8 %) were heterosexuals and 3/72 (4.2 %) were MSM.

When considering only the 1,764 subjects incarcerated during the month prior to the survey, HIV-test was offered to 1,338 (75.8 %) at prison entry and 604 (45.1 %) of these accepted to be tested, 417 (31.2 %) refused, 276 (20.6 %) were waiting for the results and 41 (3.1 %) were not tested due to their release. Among the 604 tested inmates, 20 (3.3 %) were HIV-Ab positive.

### Viro-immunological characteristics of treated patients

All the institutions provided information regarding access to viro-immunological parameters while in prison. Viral load measurement was performed in all of them, lymphocytes subsets immunophenotyping in 94.3 % and genotypic testing for HIV in 74.3 %.

An evaluation of the clinical data of 338 HIV-infected inmates, showed that 81.4 % were receiving an antiretroviral treatment (ART) and that 73.5 % showed an undetectable HIV-RNA (<50 copies/mL).

Fewer than 10 % of the detained patients on therapy, had CD4 lymphocyte counts below 200/mm^3^ (9.4 %), 18.2 % had between 200/mm^3^ and 349/mm^3^, 32.0 % between 350/mm^3^ and 500/mm^3^, and 36.4 % above 500/mm^3^. CD4 lymphocyte count was not available in 4 % of the inmates. By contrast, among the 67 inmates (19.8 %) who were not on ART, less than 5 % of the prisoners had CD4 lymphocyte counts below 200/mm^3^ (4.5 %), 15.0 % between 200/mm^3^ and 349/mm^3^, 10.4 % between 350/mm^3^ and 500/mm^3^, and 70.1 % had CD4 above 500/mm^3^. CD4 lymphocyte count was not available for 4 % of these patients.

### HIV treatment and care

The majority of known HIV-positive inmates (275/338; 81.4 %) were receiving antiretroviral treatment at the time of the study. Of them, 172/275 (62.5 %) were receiving a protease inhibitor (PI)-based ART, 72 (21.0 %) a non-nucleoside reverse transcriptase inhibitors (NNRTIs)-based ART and 27 (9.8 %) raltegravir as an integrase inhibitor-based ART. The difference in the proportion of patients treated patients with PI-based regimens compared to those receiving NNRTIs-based regimens was statistically significant (*p* < 0.005).

PI-based antiretroviral regimens included atazanavir in 88 (32.0 %) patients, lopinavir (18.5 %) in 51, darunavir in 23 (8.4 %), fosamprenavir in 9 (3.3 %) and saquinavir in 1 (0.4 %), as shown in Table 2.

NNRTI-based ART regimens included efavirenz in 50 (18.2 %) patients, nevirapine in 9 (3.3 %), rilpivirine in 8 (2.9 %), and etravirine in 5 (1.8 %). In 41/50 (82 %) patients efavirenz was included in the efavirenz-tenofovirdisoproxilfumarate-emtricitabine co-formulation and in 6/8 (75 %) rilpivirine was part of the rilpivirine-tenofovirdisoproxilfumarate-emtricitabine co-formulation (Table 2).

The backbone in the 275 HIV-positive inmates consisted of 194 (70.5 %) tenofovirdisoproxilfumarate-emtricitabine, 41 (14.9 %) abacavir-lamivudine, 12 lamivudine-zidovudine (4.4 %), and 28 (10.2 %) other NRTI combinations (Table 2).

The medical visit schedule was on weekly basis in 4.3 % of prisoners under ART, on monthly basis in 32.9 %, on quarterly basis in 51.4 % and every 6 months in 11.4 %.

All the correctional institutes had medical facilities able to dispense antiretroviral drugs. Lack of HIV treatment in prisoners with CD4 lymphocyte count < 350/mm^3^ was due to medication refusal (69.2 %), ongoing medical assessment (23.1 %) and other reasons including fear of side effects, cultural or ethnic beliefs, depression or delusional state (7.7 %).

Medication dispensing by nurse practitioners to those who were under ART occurred in prison (70 %) or at the medical unit (30 %). The methods for dispensing medications were: daily directly observed therapy (DOT, 42.8 %), keep-on-person (KOP, 31.4 %) with daily delivery and a mixed combination (25.7 %). Frequency of dispensing was once a day (17.2 %), twice a day (31.4 %) or ≥ 3 times a day (51.4 %). Once a day regimens were perceived to facilitate DOT by a majority (65.7 %) of the healthcare staff with only 25.7 % considering these regimens useless to DOT.

After prison release, nearly two-thirds (66.4 %) of the prisoners were followed up by infectious disease specialists.

## Discussion

This study was designed to provide a current picture of HIV prevalence, treatment and care in the Italian prison population.

The lack of a comprehensive surveillance system makes it difficult to estimate the prevalence of HIV-infected inmates in the Italian correctional system. Published prevalence data are usually taken from studies conducted on small numbers of prisons or in single regions, this hampering the achievement of definite data. Likewise, little is known about treatment of HIV among inmates [7, 10, 11, 14]. Medical assistance to HIV-infected individuals is a relevant issue in the community of inmates and treatment monitoring is essential to guarantee an appropriate use of antiretroviral drugs and high quality patient care [7, 10, 15–18]. Point-prevalence studies based on existing medical data in correctional institutes could help to better understand the spread of HIV as well as elaborate successful prevention and care strategies.

Although our findings are limited by the partial participation of the Italian penal institutes, they contribute to improve our knowledge on HIV infection, treatment and care among HIV-patients detained in Italy. In fact, with more than 15,000 inmates in 35 prisons distributed all over the country, our investigation includes nearly one-quarter of those incarcerated in Italy, with 27.0 % being IVDU and 37.1 % being foreigners at the time of the study.

Looking at the distribution of the participating prisons across the country and at the composition of HIV-risk factors among inmates, our data seems to be reasonably representative of the Italian prison population.

Despite the low rate of HIV testing identified in our study and the well-known difficulty in inmate patients to admit their disease condition, we found an HIV prevalence above 5 % which is more than 12 times higher than in the general Italian population [19, 20].

This discrepancy was also confirmed in newly admitted prisoners (HIV testing rate 34.2 %, HIV prevalence 3.3 %) and underline that prisons are concentrators of infectious diseases, due also to higher risk behaviours in acquiring blood born viruses (BBV) in people likely to be incarcerated. In our study we show an high rate of IVDU and tattoing/body piercing among both Italian and foreign BBV-infected inmates. For these reasons, the imprisonment period should be considered as an opportunity to treat a hard-to-reach population in freedom [21–27]. Finally, we cannot exclude the possibility that the real prevalence of HIV infection might have been underestimated by the low rate of testing.

Some findings deserve attention in our study. Firstly, foreign detainees represent 37.1 % of prisoners, the vast majority coming from low and middle-income countries where the health burden imposed by HIV is further exacerbated by poor socioeconomic conditions, the high prevalence of opportunistic infections, poor access to health care, and widespread drug circulation and abuse. Secondly, Italian prison population contains nearly one-third of prisoners with a history of drug misuse at some time in their lives. Given the interplay between transmissible diseases, drug use, low-to middle-income countries origin and incarceration, there is a need to develop approaches to increase the acceptance of testing by raising an awareness in prisoners regarding infections, Appropriate testing pathways in prison should be optimized to ensure adequate pre- and post-test discussion, and to develope care pathways that enable treatment in prison as well as continuity of care upon release [28–32]. It is essential that prison healthcare personnel, in particular infectious disease specialists, make every effort to increase the offer of HIV screening in prison; indeed, the knowledge of HIV status among inmates is the only condition for HIV prisoners to access antiretroviral drugs and to obtain continuity of care when released [31, 32].

Moreover, we found a high proportion of patients with concomitant chronic viral hepatitis caused by HBV in 6.8 % and by HCV in 55.9 %. Patients with HBV coinfection should start antiretroviral therapy active also against HBV. The very high proportion of patients with HCV coinfection could represent a probably unique option to treat these patients. With the introduction of all-oral anti HCV directly-acting antiviral (DAA) drug combinations that eliminate interferon and its side effects, treatment uptake outside the prison setting is rapidly increasing. Some of these combination regimens have achieved sustained virologic response (SVR) in more than 90 % of some subgroups in clinical trials, including null responders to prior interferon-based treatment [33, 34]. Current evidence suggest that HIV/HCV co-infected can achieve the same percentage of response as HCV mono-infected [35]. The prison setting, with the possibility of DOT also for HCV, could represent a key option in order to obtain HCV eradication and reduce the progression of liver disease for the single patient as well as reducing the chance of transmission inside or, outside prison, after release.

Even in a prison setting, standard-of-care strategies have showed that health outcomes among HIV-infected inmates improve significantly. It is well established that the availability of combination antiretroviral therapy in prison is largely responsible for decreased AIDS-related mortality and morbidity among inmates in the recent years in high-income countries [33–38]. Our study shows that the vast majority of the known HIV-infected inmates were under therapy often taken with daily DOT. Successful HIV suppression was shown in over 72 % of treated patients who had an undetectable HIV-RNA. Nevertheless, several obstacles to HIV treatment in prison still remain. High costs, difficulties in maintaining confidentiality, lack of trust in correctional staff as well as the social dynamics of correctional facilities are all implicated as barriers to HIV treatment, as suggested by our findings.

We found that almost two out of three of the treated prisoners were given PI-based regimens compared to nearly one in four who received NNRTIs-based combination. Most patients probably then continue the regimens they receive in the community setting. The choice of PI-based HAART is probably due to the perceived low adherence by the physician in freedom and the high proportion of PI-based schedules is the continuation of ongoing treatment. However, when considering organization problems in prison, together with the need to treat prisoners with complex behaviors in a problematic context, caregivers should aim at reducing pill burden and dosing frequency [32, 37, 39–42]. The availability of single tablet regimens with good forgiveness can be an option that combines efficacy, safety and low pill burden giving the opportunity to the patients to continue such combinations even after incarceration [39–49]. It is thus mandatory to remember that regimen simplification can be implemented only if the suppression of HIV-RNA is ensured. Simplification can be a useful approach not only to reach successful viro-immunological outcomes among prisoners under HIV treatment, but also to improve the patient’s quality of life, maintain long-term adherence, avoid toxicities that may develop with prolonged ART and reduce the risk of virologic failure [50–52].

Although we found that over 80 % of HIV-infected inmates were treated for HIV with more than 70 % with undetectable viral load in the Italian correctional institutes, this finding does not account for the needs of infected unaware prisoners. Nearly 67 % of patients not receiving HAART at the time of the study had a CD4 cell count <350 cells/mm^3^, which represents the recognized threshold for therapy initiation in all international guidelines. This data highlights the need for the physician working in prison to be more proactive in convincing patients to start HAART since there is a clear benefit in term of morbidity and mortality reduction [36–40]. The persistence of unprotected sexual relations as well as the injection of drugs without sterile equipment or with needle sharing during incarceration strengthens even more the need to test and treat HIV-infected inmates in order to reduce virus transmission, as observed in other settings [53–57].

Upon release only approximately two-third of the prisoners were followed up by infectious disease specialists. The loss to follow up of one-third of patients highly stresses the need to integrate HIV prevention and treatment services both outside and within correctional institutions. The integration of care should include access to medical discharge planning and referral to community-based HIV care providers, both being of utmost importance to guarantee continuity of care when inmates are released back into the community [58–64].

Treating HIV-infected inmates poses significant challenges, but there are several obstacles to the proper intake of anti-HIV drugs, not only due to patients. Prison doctors may be wary of managing complicated treatment regimens which often have adverse side effects, especially in high-risk populations such as IVDU. Inadequate prison infrastructures are a significant barrier to implementing comprehensive HIV care in Italy. Problems occur with patient non compliance, medical contraindications and high medication costs. Finally, inmates may be reluctant to seek testing and treatment because of fear, denial or distrust of the competence of correctional medical staff.

## Conclusions

HIV treatment in Italian prisons is not uniform and this undermines the ability to provide high-quality care for the inmates infected with HIV. Diagnostics, treatment and care are offered to the majority of HIV-infected inmates, but the costs of not treating a part of this population could be significantly higher. Prisons should represent an integral part of strategies to slow down the HIV and possibly the HCV epidemic through the successful treatment of infected inmates and missed opportunities for treatment could have negative consequences not only on the incarcerated population, but on society as a whole. Therefore, nationwide programs, integrated with the National Health System, should be implemented to increase the quality of care in Italian prisons and encourage linkage to care after prison release.

## Abbreviations

SIMSPe: Società Italiana Malattie e Sanità Penitenziaria
SIMIT: Società Italiana di Malattie Infettive e Tropicali
HIV: human immune deficiency virus
NNRTI: non nucleoside reverse transcriptase inhibitor
NRTI: nucleoside reverse transcriptase inhibitors
PI: Protease inhibitor
HBV: Hepatitis B Virus
HCV: Hepatitis C Virus
ART: antiretroviral therapy
DOT: daily directly observed therapy
KOP: Keep-on-person therapy
IVDU: intravenous drug users
DAA: directly-acting antiviral
SVR: sustained virologic response
AIDS: acquired immune deficiency syndrome
